# Multidrug-resistant tuberculosis outbreak associated with poor treatment adherence and delayed treatment: Arua District, Uganda, 2013–2017

**DOI:** 10.1186/s12879-019-4014-3

**Published:** 2019-05-07

**Authors:** Denis Okethwangu, Doreen Birungi, Claire Biribawa, Benon Kwesiga, Stavia Turyahabwe, Alex R. Ario, Bao-Ping Zhu

**Affiliations:** 1 Uganda Public Health Fellowship Program, Kampala, Uganda; 2 National Tuberculosis and Leprosy Program, Ministry of Health, Kampala, Uganda; 3 US Centers for Disease Control and Prevention, Kampala, Uganda; 4 Division of Global Health Protection, Center for Global Health, US Centers for Disease Control and Prevention, Atlanta, GA USA

**Keywords:** Multidrug-resistant tuberculosis, Rifampicin, Isoniazid, Global health security

## Abstract

**Background:**

In August 2017, the Uganda Ministry of Health was notified of increased cases of multidrug-resistant tuberculosis (MDR-TB) in Arua District, Uganda during 2017. We investigated to identify the scope of the increase and risk factors for infection, evaluate health facilities’ capacity to manage MDR-TB, and recommend evidence-based control measures.

**Methods:**

We defined an MDR-TB case-patient as a TB patient attending Arua Regional Referral Hospital (ARRH) during 2013–2017 with a sputum sample yielding *Mycobacterium tuberculosis* resistant to at least rifampicin and isoniazid, confirmed by an approved drug susceptibility test. We reviewed clinical records from ARRH and compared the number of MDR-TB cases during January–August 2017 with the same months in 2013–2016. To identify risk factors specific for MDR-TB among cases with secondary infection, we conducted a case-control study using persons with drug-susceptible TB matched by sub-county of residence as controls. We observed infection prevention and control practices in health facilities and community, and assessed health facilities’ capacity to manage TB.

**Results:**

We identified 33 patients with MDR-TB, of whom 30 were secondary TB infection cases. The number of cases during January–August 2017 was 10, compared with 3–4 cases in January–August from 2013 to 2016 (*p* = 0.02). Men were more affected than women (6.5 vs 1.6/100,000, *p* < 0.01), as were cases ≥18 years old compared to those < 18 years (8.7 vs 0.21/100,000, *p* < 0.01). In the case-control study, poor adherence to first-line anti-TB treatment (aOR = 9.2, 95% CI: 2.3–37) and initiating treatment > 15 months from symptom onset (aOR = 11, 95% CI: 1.5–87) were associated with MDR-TB. All ten facilities assessed reported stockouts of TB commodities. All 15 ambulatory MDR-TB patients we observed were not wearing masks given to them to minimize community infection. The MDR-TB ward at ARRH capacity was 4 patients but there were 11 patients.

**Conclusion:**

The number of cases during January–August in 2017 was significantly higher than during the same months in 2013–2016. Poor adherence to TB drugs and delayed treatment initiation were associated with MDR-TB infection. We recommended strengthening directly-observed treatment strategy, increasing access to treatment services, and increasing the number of beds in the MDR-TB ward at ARRH.

## Background

Multidrug-resistant tuberculosis (MDR-TB), caused by *Mycobacterium tuberculosis* resistant to at least isoniazid and rifampicin, is an increasing global public health problem [1]. Strains resistant to anti-TB treatment may be transmitted from person to person, or resistance may be acquired during standard TB treatment during quinolone treatment for other conditions, inappropriate TB treatment, or poor adherence to treatment [2]. Though a shorter course of treatment has been approved by the World Health Organization, treatment for MDR-TB still typically lasts about 2 years, with a 55–67% cure rate; in comparison, a standard 6-month treatment regimen can achieve a 95% cure rate for drug-susceptible TB cases [3, 4]. The prolonged treatment for MDR-TB requires expensive second-line regimens and presents serious financial challenges for national TB programs and families, especially in the resource-limited sub-Saharan Africa. In many countries, treatment of MDR-TB consumes more than half of the national TB control program budget and threatens the effectiveness of national TB control programs [5]. The World Health Organization (WHO) estimates that in 2016, there were 600,000 incident cases of drug-resistant TB globally, including rifampicin-resistant-tuberculosis [6]. This represents a rise from the previous year, in which there were an estimated 580,000 incident drug-resistant-TB patients, 100,000 of whom had rifampicin-resistant TB. During the same year, 250,000 people were estimated to have died from MDR-TB, mostly in Asia [7].

A 2013 study suggested that the proportion of TB cases that were drug-resistant in sub-Saharan Africa is lower than in some countries in Eastern Europe and Asia [2, 6]. However, the burden of MDR-TB in sub-Saharan Africa is not well known because of poor surveillance and diagnostic capabilities [8]. In Uganda, the prevalence of MDR-TB in 2015 was estimated to be 1.6% (0.78–2.4) among newly diagnosed TB cases and 12% (3.4–21) among previously-treated TB cases [9].

Socio-political instability can increase the risk for emergence of MDR-TB [4]. Persons seeking refuge from unstable areas, including refugees, rejected asylum seekers, and victims of trafficking may be at a particularly high risk due to interruption of TB drug supply and exposure to MDR-TB-infected persons during transit [10]. In 2016, there was a large influx of refugees from South Sudan into Uganda. Data on the MDR-TB situation in South Sudan are not readily available; however, one study conducted in South Sudan appeared to indicate an increasing number of cases from 2004 to 2008 [11]. Most of these South Sudanese refugees settled in the West Nile districts in Northwestern Uganda; one of these is Arua District, which accommodates refugees in Rhino Camp and Imvepi settlements.

Of the eight districts in the West Nile sub-region of Uganda, seven have a GeneXpert machine for diagnosis of rifampicin-resistant TB. Patients diagnosed with rifampicin-resistant TB from the sub-region are referred to Arua Regional Referral Hospital (ARRH), where MDR-TB treatment is initiated while samples are referred to the National Tuberculosis Reference Laboratory for drug susceptibility testing (DST) to confirm multidrug-resistance. The GeneXpert was first deployed in the West Nile region in 2012; this was followed by another in, 2013, 2014, and in 2017 (National TB and Leprosy Control Program, 2017 unpublished). In Arua District, there was no extra deployment of a Genexpert machine since 2013.

In August 2017, Medical Teams International, a Non-Governmental Organization that operates in the region among refugees, reported to the Ministry of Health of Uganda that the number of MDR-TB cases in West Nile appeared to be increasing, including confirmed cases among refugees in settlements in Arua District. We conducted an investigation to confirm the reported upsurge in cases, identify risk factors for developing MDR-TB, assess the capacity of health facilities in Arua District to diagnose and manage TB, and recommend control and prevention measures.

## Methods

### Study design

This was an outbreak investigation. We followed the key steps of disease outbreak investigations as described by the US Centers for Disease Control and Prevention [12].

### Study area

Arua District, Uganda (3.0303299 N, 30.9073040) is located in northwestern Uganda. It has an estimated population of 840,900 (Uganda Bureau of Statistics, unpublished). The district comprises 28 sub-counties, three of which host refugees (Rhino Camp, Oluko and Rigbo sub-counties). In 2017, the sub-region had an HIV prevalence of 3.1% [13].

### Case definition

We defined a TB case as one or more positive sputum smears or cultures since 2013 in a person residing in Arua District with clinical features consistent with TB. An MDR-TB case-patient was a person diagnosed with TB, with a sputum sample yielding *M. tuberculosis* that was resistant to at least rifampicin and isoniazid, confirmed by culture, line probe assay, or any other drug susceptibility test since January 2013 to August 2017.

### Data abstraction and case finding

Using registers obtained from ARRH, we identified all patients who were diagnosed with TB and MDR-TB from ARRH. From their medical records, which were retrieved from the same facility, we abstracted data on those TB and MDR-TB cases who presented during January 2013–August 2017 at the TB initiation center. Patients with MDR-TB were included in the study only if they had results from a drug susceptibility test. Clinically-diagnosed patients and those without test results were excluded from the study. Data abstracted included TB and MDR-TB case-patients’ age, sex, occupation, and place of residence. With the help of the Village Health Team and community key informants, we traced and interviewed each MDR-TB case-patient to collect information on exposure risk factors. These factors were smoking status, alcohol use, self-reported adherence to first-line TB and MDR-TB drugs, reasons for non-adherence, date of initial diagnosis with TB, date they developed symptoms of TB, presence of any co-morbidities, refugee status, type and status of housing, household size, primary or secondary MDR-TB, and contact with anyone diagnosed with TB before they were diagnosed with the disease. Non-adherence to TB treatment was defined as missing at least 10% of total prescribed drugs in a specified period [14].

### Descriptive epidemiology

We conducted a descriptive analysis of MDR-TB case-patients by time, person, and place. Using open-ended questionnaires, we conducted hypothesis-generating interviews with case-patients to gather data on exposure status, date of onset of TB signs and symptoms, date of diagnosis, and commencement of first-line anti-TB treatment, self-reported adherence to TB treatment, HIV status, self-reported adherence to antiretroviral therapy (ART) if HIV-positive and on therapy, smoking, alcohol use, and refugee status.

### Case-control study

We conducted a case-control study during 24–28 August 2017, using MDR-TB cases identified from the review of health facility records. A control was a TB case-patient in Arua District who had been on anti-TB treatment for at least 2 months, enrolled on treatment within the same period as the case, and with negative GeneXpert results for drug resistance during the course of their treatment. A sampling frame for controls was prepared using health facility records. We used systematic random sampling to select three controls for every case, matched by sub-county of residence.

### Assessing infection prevention and control practices

We assessed adherence to IPC practices in health facilities and the community by using a checklist and observing the practices. Indicators assessed included infrastructure adequacy, availability and use of N95 respirators by healthcare workers, and surgical masks by TB patients.

### Assessment of health facilities for capacity to diagnose and manage TB

We purposefully selected ten health facilities from a total of 84 health facilities in the district, including ARRH, and assessed them for capacity to diagnose and manage TB patients. The selected facilities were health facilities that followed up MDR-TB patients who were discharged from ARRH after the intensive phase of treatment. We assessed each facility with the following indicators: number and skill mix of staff, whether they had received any MDR-TB or HIV/TB co-morbidity training, diagnostic infrastructure (including availability of functional microscopes, slides, and GeneXpert machine), and whether they screened for TB at all service points within the facility. Other variables assessed included whether TB patients were on DOTS verified through the records in the health facilities, availability of reliable transportation for sample referrals for GeneXpert diagnosis, and history of stockout of essential TB commodities (e.g., slides, TB drugs and GeneXpert panels).

### Statistical analysis

Using surveillance data, we estimated the prevalence of TB in Arua District using the district population as the denominator. Population data were obtained from Uganda Bureau of Statistics projections (unpublished) for 2015–2020 and National Housing and Population Census report, 2014 [16]. We also disaggregated TB cases by sub-county, from which we computed the proportion of TB cases that was MDR-TB by sub-county, using sub-county population data obtained from the district headquarters (unpublished). We drew maps of Uganda and Arua District with its sub-counties using QGIS software (QGIS Development Team, 2009. QGIS Geographic Information System, Open Source Foundation Project. http://qgis.osgeo.org). To determine whether the reported number of MDR-TB cases during January–August in 2017 was a significant aberration from the expected number of cases, we calculated the mean number of cases each year during January–August 2013 to 2017 and estimated the Poisson probability of observing at least the number of reported cases in 2017 [17]. This was because we did not have data from September–December 2017 at the time of this report. We evaluated the association between MDR-TB and exposure risk factors using conditional logistic regression at bivariate and multivariable levels. Our multivariable model comprised all variables that were significant at the bivariate analysis. In the regression model, we created a variable called ‘unknown’ to adjust for missing data.

### Ethical considerations

Ethical clearance for the investigation was obtained from the Uganda Ministry of Health and from the U.S. Centers for Disease Control and Prevention (CDC), where the evaluation was deemed non-research. Permission was also obtained from the Arua District Health Office; ARRH granted permission to access their data. Health facility administrators gave us permission before any assessments of their facilities were conducted. Before analysis, we removed all identifying data and assigned a study identification number to ensure anonymity.

## Results

### Descriptive epidemiology

**Fig. 1:**
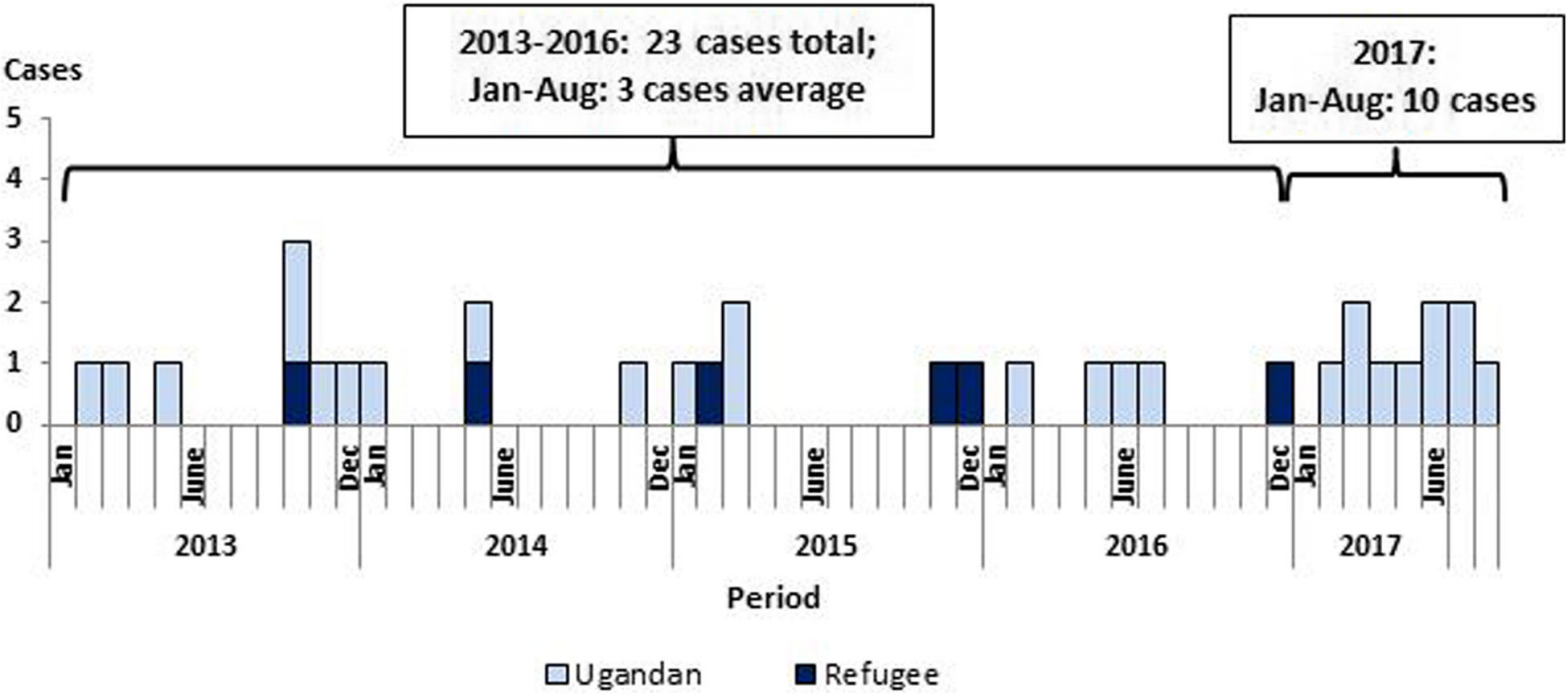
Distribution of multidrug-resistant tuberculosis cases by month: Arua District, Uganda, 2013–2017

We identified 33 MDR-TB cases from Arua District enrolled for treatment from February 2013 to August 2017 **(**Fig. 1**)**. Three of the cases (9.1%) of the cases were primary MDR-TB cases. The median age of the 33 case-patients was 42 (range: 10–67) years; 26 (79%) were male and 32 (97%) were ≥ 18 years. The average annual population incidence over the 5 years was 0.82 cases per 100,000 population. During January–August 2017, there were 10 MDR-TB cases, compared with three cases during the same 8 months each year in 2013 to 2015, and four in 2016 (*p* = 0.0195). The average monthly number of MDR-TB cases was 1.3 in 2017, compared with 0.46 during 2013–2016. Considering the district population, the average annual incidence of MDR-TB among men was higher than that among women at (6.5 vs 1.6/100,000) (*p* < 0.001). Among refugees, the average annual MDR-TB incidence was 2.8/100,000 (6 cases), compared to the average annual incidence among Ugandan nationals of 3.2/100,000 (27 cases) (*p* = 0.9) **(**Table 1**)**.

**Table 1:** Population incidence of multidrug-resistant tuberculosis by socio-demographic characteristics: Arua District, 2013–2017

Characteristic	Frequency (*n* = 33)	Population	Population Incidence (/100,000)
Age (years)			
< 18	1	471,030	0.21
≥ 18	32	369,870	8.7
Sex			
Male	26	400,281	6.5
Female	7	440,619	1.6
Nationality status			
Refugee	6	211,749	2.8
Ugandan	27	840,900	3.2

**Fig. 2:**
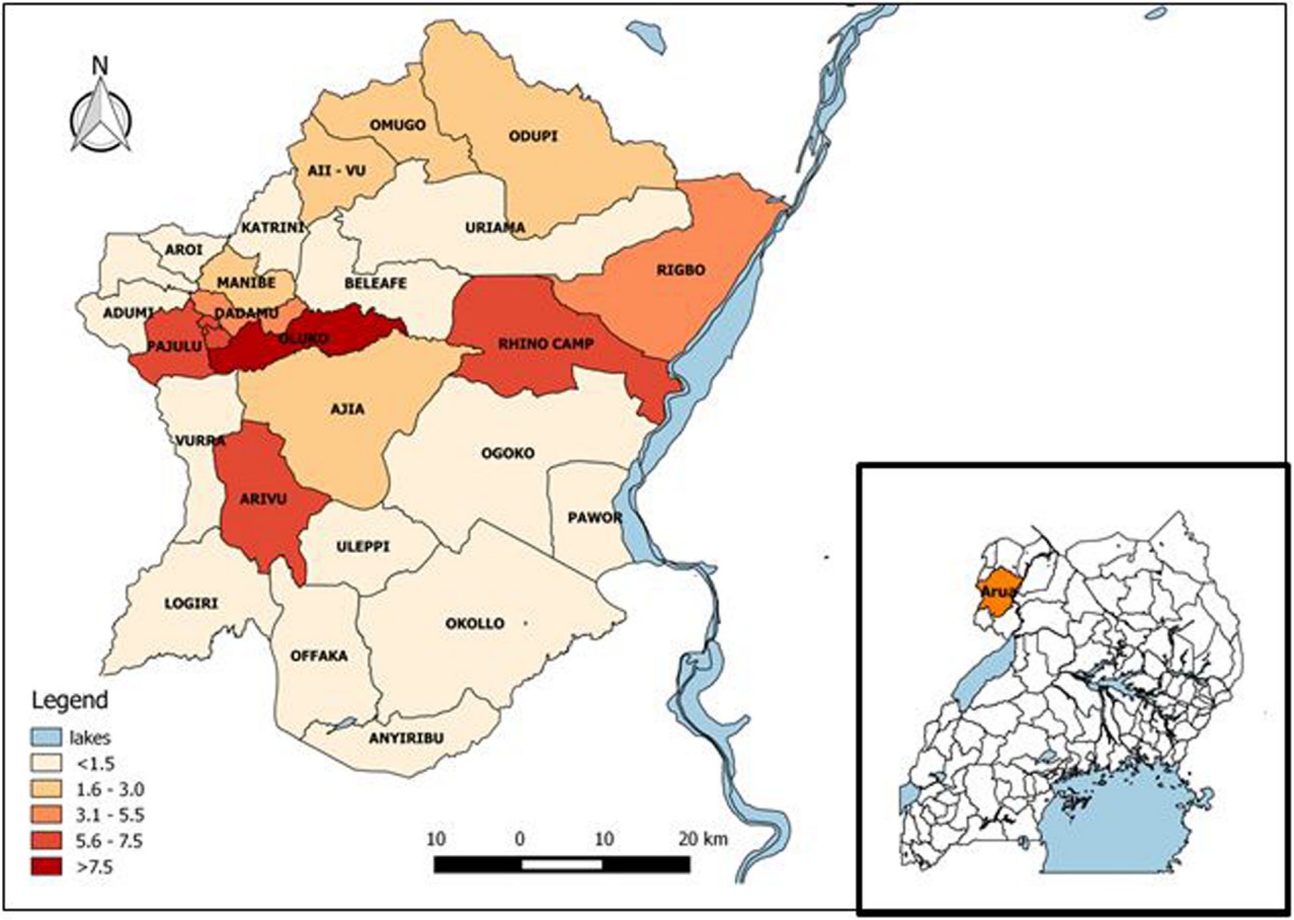
Proportion of tuberculosis cases that were multidrug-resistant tuberculosis (per 1000) by sub-county: Arua District, Uganda, 2013–2017: Inset: map of Uganda (Drawn by author using QGIS software)

During 2013 to 2017, there were 4742 TB reported cases in Arua District (District TB and Leprosy Supervisor’s report 2017, unpublished). Hence, the 5-year proportion of all TB cases that was MDR-TB was 0.70 /1000 (33 cases). The 33 MDR-TB patients came from 48% (13/27) of sub-counties in Arua District. Of the affected sub-counties, the proportion of all TB cases that were MDR-TB was highest in Oluko sub-county (0.21/1000) and lowest in Omugo sub-county (0.023/1000) **(**Fig. 2**)**.

### Case-control study findings

Non-adherence to first-line TB treatment occurred in 45% (15/33) of case-patients compared with 9.9% (10/101) of controls (OR = 11, 95% CI: 3.9–33). Similarly, among the case-patients, 12% (4/33) initiated treatment ≥15 months after the start of TB symptoms, compared with 5% (5/101) among controls (OR = 6.5, 95% CI: 1.3–32). Also, 52% (17/33) of case-patients compared with 32% (33/101) of controls had HIV co-infection (OR = 2.6, 95% CI: 1.1–6.1). Contact with another TB patient, smoking, drinking alcohol, refugee status, and occupation were not significantly associated with development of MDR-TB.

In the multivariable conditional logistic regression analysis, non-adherence to first-line anti-TB treatment (aOR = 9.2, 95% CI: 2.3–37) and delayed treatment initiation ≥15 months after start of TB symptoms (aOR = 11, 95% CI: 1.5–87) were significantly associated with MDR-TB **(**Table 2**)**.

**Table 2:** Factors associated with infection with multidrug-resistant tuberculosis: Arua Regional Referral Hospital, Uganda, 2013–2017

Exposure	% Cases (*n* = 30)	% Controls (*n* = 92)	OR (95% CI)	*p*-value	aOR^a^ (95% CI)	*p*-value
Adhered to first-line TB treatment						
Yes	33	89	Ref		Ref	
No	47	11	11 (3.5–33)	< 0.01^#^	12 (1.9–79)	0.009^#^
Unknown	20	0	–	–		
Mean symptomatic duration (months) before treatment						
≤ 4	17	71	Ref			
5–9	3.3	14	0.82 (0.081–8.4)	0.87	1.0 (0.086–13)	0.98
10–14	6.7	9.8	1.9 (0.29–14)	0.51	2.3 (0.22–24)	0.48
15–192	13	2.2	21 (2.7–166)	0.004^#^	24 (1.7–353)	0.02^#^
Unknown	60	3.3	45 (11–183)	< 0.01	33 (3.8–201)	< 0.01
HIV status						
Negative	47	68	Ref		Ref	
Positive	53	32	2.6 (1.1–6.1)	0.033^#^	1.3 (0.24–7.0)	0.77
Contact						
No	30	38	Ref			
Yes	63	38	2.1 (0.78–5.6)	0.14		
Unknown	6.7	24	0.27 (0.047–1.5)	0.14		
Alcohol drinker						
Never	50	41	Ref			
Former	20	35	0.49 (0.17–1.4)	0.18		
Yes	30	24	1.1 (0.39–2.9)	0.90		
Smoker						
Never	47	50	Ref			
Former	33	24	1.5 (0.60–3.9)	0.37		
Yes	17	26	0.66 (0.21–2.1)	0.48		
Unknown	3.3	0	–	–		
Nationality status						
Ugandan	83	82	Ref			
Refugee	17	18	0.84 (0.26–2.7)	0.77		

^#^Statistically significant variables

^a^aOR = adjusted odds ratio using conditional logistic regression

### Assessment of health facilities for diagnostic and management capacity

Of the 10 facilities assessed, 8 (80%) had an accredited laboratory that could provide TB diagnostic services. All reported having a functional microscope in their laboratories. All facilities reported having experienced stockouts of TB drugs, microscope slides, laboratory reagents, and N95 respirators at some point in the period from 2013 to 2017. ARRH reported that they had experienced stockouts of GeneXpert cartridges. During our visit, all facilities had drugs in stock; however, only ARRH had any N95 respirators. While all accredited facilities reported having a functional laboratory, only 5 (63%) had at least two laboratory staff; 4 (50%) reported not having any clinical staff trained in MDR-TB management **(**Table 3**)**.

**Table 3:** Health facility assessment during multidrug-resistant tuberculosis investigation: Arua District, 2013–2017

Area of assessment	% (*n* = 9)
Health facility accreditation for TB services	89
Staffing levels	
Laboratory	89
Clinicians	100
Dispensers	33
Lab availability	89
GeneXpert availability onsite^a^	100
Clinical staff trained in:	
TB/HIV	89
MDR-TB	56
Registers available	
Unit TB register	100
Laboratory TB register	56
Presumptive TB register	33
MDR-TB register	11
Transportation access	89
Mean turnaround time for GeneXpert results (weeks)	2
Stock-outs	
Anti-TB drugs	100
Microscope slides	100
Reagents	100
Masks	100
VN95 respirators	100
GeneXpert catridge^a^	100
Microscope available	89

^a^The denominator for these variables is 1. Only ARRH had a Genexpert onsite

### MDR-TB infection control and prevention

During our visit in August 2017, there were 11 inpatients with MDR-TB; however, the MDR-TB isolation ward was built to accommodate only four inpatients. This forced some MDR-TB patients to be shifted to a ward outside the MDR-TB isolation area that was designed to accommodate drug-susceptible TB patients. In all 10 health facilities evaluated, no personnel wore masks when interacting with TB patients. However, ventilation was adequate as windows in all the facilities were left open. In the community, no ambulatory MDR-TB case-patients were wearing masks given to them at discharge from ARRH or any protective masks to minimize transmission. Additionally, in over 80% of homes visited, windows were not open. MDR-TB patients shared sleeping rooms with members of their household without any precautionary measures in place.

## Discussion

The average monthly number of incident MDR-TB cases in 2017 in Arua District was more than twice those during 2013–2016. Poor adherence to first-line TB drugs and delayed treatment initiation relative to the onset of signs of TB were associated with development of MDR-TB. Of the 10 health facilities investigated, all reported stockouts of essential TB supplies at some point during the study period from 2013 to 2017 and all had inadequate numbers of trained laboratory personnel. Nearly half of healthcare workers were untrained in MDR-TB management. The MDR-TB treatment initiation center was overcrowded, forcing the hospital leadership to transfer some MDR-TB patients to the regular TB ward, increasing the risk of transmission. In the communities, ambulatory MDR-TB patients did not wear face masks provided to them at discharge from ARRH as they proceed to the stabilization phase of treatment. They therefore mix freely with community and household members without any precautions. Multiple studies have found that obligatory wearing of facemasks reduced transmission of MDR-TB by 56%, though inadequacy in its use in isolation has been highlighted [16–18].

Poor adherence to first-line TB drugs may have been caused by both patient and institutional factors. Our investigation showed that health facilities managing TB and MDR-TB cases sometimes ran out of essential TB drugs, which could result in treatment interruption. On the other hand, patient-related factors, e.g., financial challenges with transport, lack of social support, forgetfulness, being away from home, alcohol use, and perceived or real side effects of drugs, have been cited as common barriers to adherence to TB treatment in African countries and other developing countries with endemic MDR-TB [15, 19]. Our findings regarding the positive association between MDR-TB and poor adherence to first-line TB treatment are corroborated by other studies conducted in sub-Saharan Africa [2]. A meta-analysis of studies conducted on MDR-TB found that patients who had inappropriate TB treatment, including those who had inadequate adherence, had a 27-fold higher risk of developing MDR-TB than those who did not [20]. Other studies have also demonstrated that the risk of development of mono-drug or multidrug-resistant tuberculosis was strongly associated with previous exposure to TB drugs, especially in inadequate or inappropriate doses [2]. Similar results were observed in studies conducted in India and Pakistan [21, 22]. Further studies should be conducted to understand more fully the specific drivers of poor adherence to TB treatment in Arua District, with the aim of planning targeted, effective strategies to address non-adherence.

Unnecessary delay in taking appropriate action may be occasioned by a long turn-around time between sample collection and release of results, and even more time before appropriate treatment is administered. Such delays in diagnosis and initiating treatment among MDR-TB patients prolongs the time spent in the community, and therefore increases the likelihood of transmitting the disease to others in their community [23]. A study conducted in China found an association between MDR-TB and delayed initiation of treatment among MDR-TB patients; reasons for delayed treatment were identified as poor knowledge about TB, financial burden, and poor accessibility to TB services [24]. In South Africa, interventions to increase awareness of TB among the population, including improving the availability of free TB services, significantly reduced delays in treatment initiation [25]. In our study, the association between delayed initiation of treatment and MDR-TB was rather surprising since spontaneous mutation, which may occur to isoniazid and rifampicin is deemed unlikely [26]. This association may therefore have been due to undocumented inadequate exposure to TB drugs before seeking care from a qualified healthcare facility. Studies in China and Thailand also found similar association between delayed initiation of treatment and MDR-TB [24, 27].

This increase in MDR-TB cases in Arua District may have been due to a multiplicity of factors. ‘First, delays in diagnosis and treatment among MDR-TB patients may have propagated transmission in the community. Beyond this, stockout of TB drugs in health facilities might have interrupted treatment among TB patients, and therefore facilitated the development of MDR-TB. Studies need to be conducted to assess the contribution of other patient factors including alcohol and poverty. Of note is the influx of refugees from South Sudan into the region in recent years. There was a sharp increase in the refugee population in West Nile in August 2016 [28]. Refugees in Uganda are not localized in a fixed location but allowed free movement. This may increase the transmission rate of MDR-TB from infected refugees, and vice versa. Studies that characterize TB to assess dominant strains in communities that host refugees, as well as transmission patterns would offer further understanding on the epidemiology of MDR-TB in the West Nile region.

Although there was no significant association between HIV infection and developing MDR-TB, studies conducted in Ethiopia and Belarus indicate that HIV co-infection is a strong independent risk factor for MDR-TB; in both studies, respondents infected with HIV were at least two times more likely to have MDR-TB than those who were HIV-negative [29, 30]. However, findings from a study in Kazakhstan, which has a low prevalence of HIV among TB patients, showed that there was no association between HIV co-infection and development of MDR-TB [23]. Also, a nationwide survey of drug-resistant TB in Uganda found no significant association between HIV and MDR-TB [31]. Nonetheless, integrated HIV/TB management is critical in managing both diseases.

## Limitations

This study had several limitations. It was not possible to ascertain whether the observed rise in MDR-TB in the West Nile region was due to the increased deployment of improved diagnostic facilities or a real increase in cases in the region. However, records from the Uganda National TB and Leprosy Control indicate that, in the West Nile region, the GeneXpert was first deployed in 2012 in Pakwach Health Center IV and Yumbe Hospital in Nebbi and Yumbe Districts respectively; this was followed by Arua Regional Referral Hospital in Arua District in 2013. This was followed by deployments in Adjumani Hospital in Adjumani District in 2014, and in Nebbi and Moyo Hospitals in Nebbi and Moyo Districts. In Koboko (Warr Health Center IV) and Zombo Districts the Genexpert was installed in 2017 (National TB and Leprosy Control Program, 2017 unpublished). A number of variables had missing values. Nonetheless, we fitted a dummy variable called unknown in the multivariate logistic regression model in order to maintain our sample size in the analysis. In our estimation of sub-county TB incidence, we used TB cases diagnosed at facilities. This may have underestimated the incidence as undiagnosed symptomatic patients were left out.

During the investigation, we attempted to mitigate the potential adverse effect of these limitations on the validity of our study findings by using tools and strategies for data collection, triangulation and frequent corroboration of information obtained during the interviews.

## Conclusions and recommendations

Reported MDR-TB cases increased in 2017 in Arua District compared with the previous 4 years. MDR-TB was associated with poor adherence to first-line TB treatment and delay in seeking treatment. The capacity of health facilities to adequately manage MDR-TB cases was compromised. Health facilities in the district experienced stockouts of essential commodities, and had an inadequate number of trained staff in MDR-TB. While increase in MDR-TB cases may also occur due to changes in the surveillance system or strengthened laboratory capacity, there was no evidence for that in this study. We recommend reducing delays in diagnosis and treatment initiation through strengthening TB diagnostics, including GeneXpert, improved TB infection control and prevention, and supporting adherence among both local and refugee populations through DOTS and other relevant strategies. Other recommendations include expanding the MDR-TB ward at ARRH, addressing logistical challenges related to drug quantification, ordering and supply, and training healthcare workers in MDR-TB management.

## Abbreviations

ARRH: Arua Regional Referral Hospital
DOTS: Directly Observed Treatment Strategy
HIV: Human Immunodeficiency Virus
IPC: Infection Prevention and Control
MDR-TB: Multidrug-resistant tuberculosis
TB: Tuberculosis
